# Secretory Carcinoma of Minor Salivary Gland in Buccal Mucosa: A Case Report and Review of the Literature

**DOI:** 10.1155/2019/2074504

**Published:** 2019-03-27

**Authors:** Durga Paudel, Michiko Nishimura, Bhoj Raj Adhikari, Daichi Hiraki, Aya Onishi, Tetsuro Morikawa, Puja Neopane, Sarita Giri, Koki Yoshida, Jun Sato, Masayuki Ono, Yoshitaka Kamino, Hiroki Nagayasu, Yoshihiro Abiko

**Affiliations:** ^1^Division of Oral Medicine and Pathology, Department of Human Biology and Pathophysiology, School of Dentistry, Health Sciences University of Hokkaido, 1757 Kanazawa, Ishikari-Tobetsu, Hokkaido 061-0293, Japan; ^2^Division of Periodontology and Endodontology, Department of Oral Rehabilitation, School of Dentistry, Health Sciences University of Hokkaido, 1757 Kanazawa, Ishikari-Tobetsu, Hokkaido 061-0293, Japan; ^3^Sapporo Oral & Maxillofacial Surgery Clinic, Sapporo, Japan; ^4^Division of Oral and Maxillofacial Surgery, Department of Human Biology and Pathophysiology, School of Dentistry, Health Sciences University of Hokkaido, 1757 Kanazawa, Ishikari-Tobetsu, Hokkaido 061-0293, Japan

## Abstract

Secretory carcinoma (SC) of the salivary gland was recently added to the fourth edition of the World Health Organization classification of head and neck tumors. Some salivary tumors, including acinic cell carcinoma, have been reclassified as SC. Most of these tumors are located on the parotid gland with very few cases reported in the minor salivary glands of the buccal mucosa. Herein, we present a case of SC of buccal mucosa, which appeared clinically as a benign lesion in a 54-year-old Japanese female patient. Histopathologically, the tumor cells presented with an eosinophilic cytoplasm with microcytic structure along with eosinophilic secretory material and hemosiderin deposit. Immunohistochemical staining revealed strongly positive staining for S100, vimentin, and mammaglobin and negative staining for DOG-1. The tumor was finally diagnosed as secretory carcinoma of the buccal mucosa. We present a review of the medical literature of SC arising from minor salivary glands. We found only 15 cases of SC of buccal mucosa out of 63 cases of SC in the minor salivary glands. They showed good prognoses and only one case of SC in the buccal mucosa exhibited local recurrence and lymph node metastases.

## 1. Introduction

Secretory carcinoma (SC) is a rare salivary gland tumor and has been recently included in the fourth edition of the World Health Organization classification of head and neck tumors [1]. It is also known as mammary analogue secretory carcinoma since initially described by Skalova A et al. in 2010 through a series of 16 cases [2]. Most of the cases of this carcinoma have been located in the parotid gland, and only some were reported in minor salivary glands [2–4]. Herein, we report a case of SC in the minor salivary gland of the buccal mucosa and present a review of the medical literature regarding this condition.

## 2. Case Presentation

**Figure 1:**
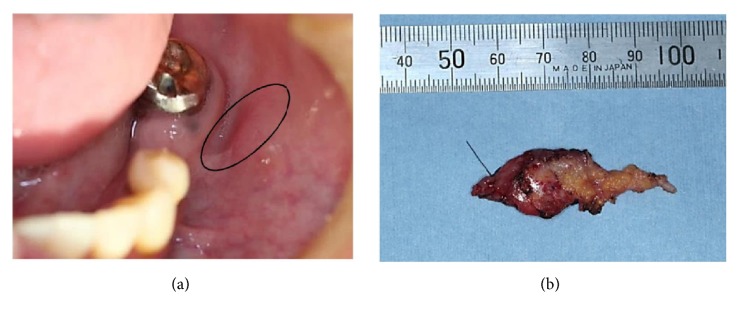
Clinical presentation of the tumor. Surgical scar on left buccal mucosa (seen inside circle) (a). The residual tumor after excision (b).

A 54-year-old Japanese female visited an oral surgery clinic with a complaint of swelling in the inner region of the left cheek for the past one month. On clinical examination, a mobile swelling (size, 1 cm x 0.75 cm) with a clear boundary was observed on the left buccal mucosa. No associated pain was reported and the overlying mucosa was normal in appearance. The swelling was clinically diagnosed as benign buccal mucosa tumor. The tumor was excised under local anesthesia and was diagnosed as acinic cell carcinoma (AcCC) after histopathological examination. The margins were still positive for the tumor and further resection was advised. The patient reported to the Health Sciences University of Hokkaido Hospital for resection of the residual tumor two months after the initial surgery. Clinically, the patient was asymptomatic. The level 1B lymph nodes on both sides were palpable, bean sized, mobile, elastic, and soft. Intraorally, a surgical scar of about 7 mm was present on left buccal mucosa. There was no pain on pressure in the region of the scar (Figure 1(a)). The patient had a history of noninvasive ductal carcinoma (ductal carcinoma in situ [DCIS]; T_is_ N_0_M_0_) in the right breast, which was treated by excision and 57 Gy of radiotherapy five months ago. On investigation for oral lesion, no obvious abnormalities were detected on the computed tomography- (CT-) scan, contrast MRI, and ultrasonogram. Positron emission tomography- (PET-) CT did not suggest transition to and from any of the distant organs. The margin was resected under general anesthesia and sent for histopathological examination (Figure 1(b)). No relation to the parotid gland was found at the time of surgery.

**Figure 2:**
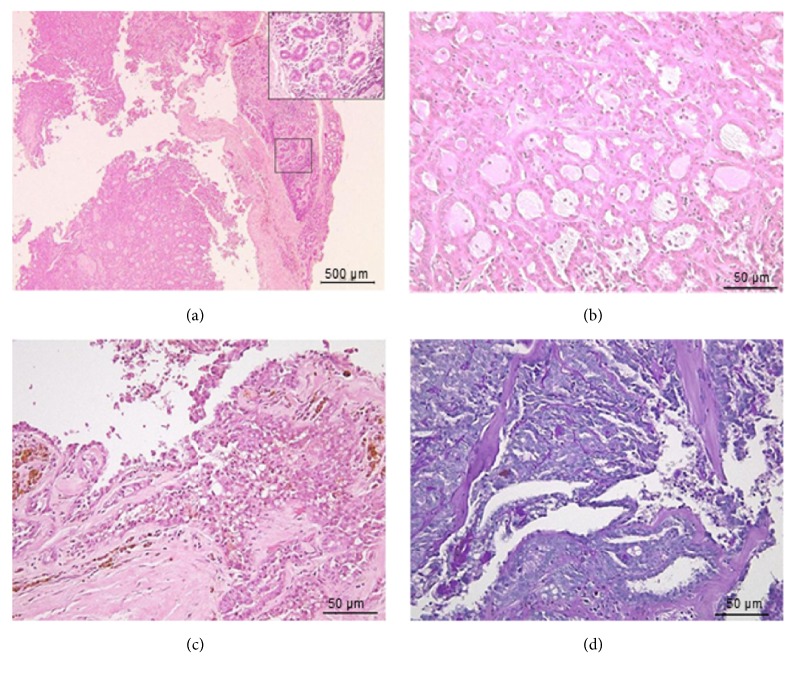
Histopathological features of SC. The excised tissue with areas of minor salivary gland (shown in inset) (a). The mass was composed of tumor cells with eosinophilic cytoplasm and had a microcystic, tubular, papillary cystic structure with eosinophilic secretory material (b, c). Few areas with hemosiderin deposition were also recognized (c). The secretory component stained positive for d-PAS (d).

Histopathologically, the excised margin appeared as a fragmented tissue with no encapsulation. The tumor tissue was composed of cells with dominant microcystic structure with eosinophilic cytoplasm and eosinophilic secretory material. Papillary and tubular pattern of cell arrangement were also found but were limited to small area. A few vacuolar cells and some areas with hemosiderin deposition were observed. Furthermore, normal muscle tissue and atrophied salivary gland tissues were also seen (Figure 2).

**Figure 3:**
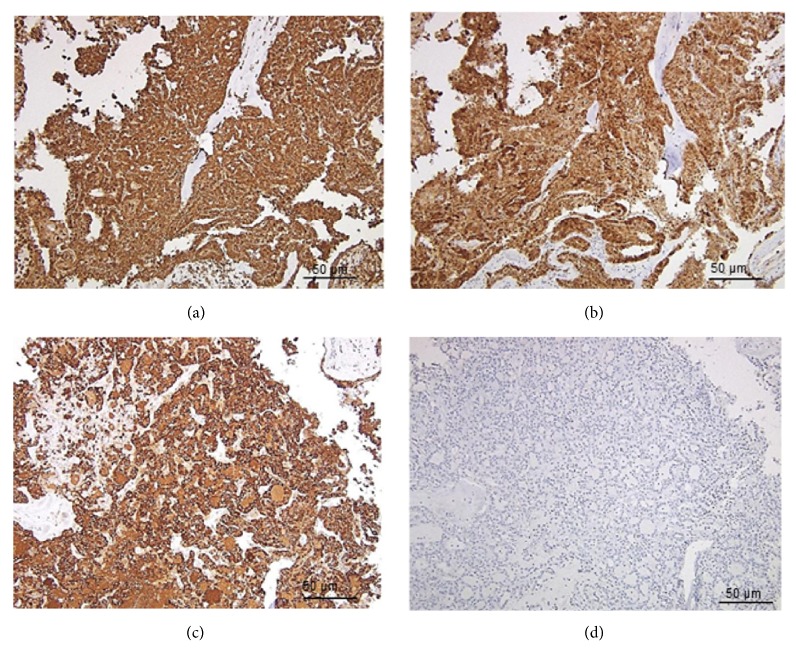
Immunohistochemical staining of the tissues. The cells were strongly positive for vimentin (a), S-100 (b), and mammaglobin (c), but negative for DOG-1 (d).

The secretory material was positive for diastase digested Periodic acid-Schiff (d-PAS), Mucicarmine, and Alcian Blue staining. No zymogen granules were found in the tumor cells. Immunohistochemistry (IHC) revealed strong positive reactions to vimentin, cytokeratin-19, and S100 protein. Mammaglobin was strongly positive, whereas discovered on gastrointestinal stromal tumors 1 (DOG-1) showed a negative reaction (Figure 3). The histological sections of breast carcinoma were examined in suspicion of metastases; however, features of ductal carcinoma in situ that appeared completely different from those of buccal mucosa tumor were noted. Based on these histomorphologic and IHC profiles, the case was diagnosed as SC of the minor salivary gland in the buccal mucosa.

## 3. Discussion

SC of salivary glands has been recently included in the fourth edition of the World Health Organization classification of head and neck tumors [1]. Since its description by Skalova et al. in 2010, some salivary tumors, including AcCC, have been reclassified as SC [2]. The majority of these cases were found in major salivary glands, with less frequency in minor salivary glands [2–4]. Our review showed that 63 cases of SC of minor salivary glands have been reported (Table 1). Among them, only 15 cases were found in buccal mucosa. The lip was the most affected site (21 cases) followed by palate (17 cases). Two cases were reported in tongue, labial mucosa, and retro molar gingiva each and 1 case in floor of mouth. The mean age of these patients was 48.1 years (range: 5-86 years). Only 2 cases were found in pediatric population [13, 18]. The sizes of the tumors ranged between 0.3 and 3.0 cm (mean 1.2cm). Among 42 cases which specified tumor size, more than half (24 cases) were of size ≤ 1 cm. Only 6 cases which were ≥ 2 cm were reported. Most of the tumors presented as a slow growing and painless mass. The only aggressive tumor was in hard palate which showed slow growth for 36 months but was aggressive for 2 months [23]. Two patients with tumor at hard palate complained of pain with ulceration [15, 22]. Lymph node metastases occurred in only 4 patients [2, 6, 23, 28] and local recurrence was reported in 4 patients [7, 23, 28]. These clinical features indicate that SC in the minor salivary glands may have a good prognosis with rare recurrence and lymph node metastases.

The present case was clinically diagnosed with benign buccal mucosa tumor. The small size of the tumor with a regular border, slow growth, normal overlying mucosa, and absence of pain suggested the lesion might be benign. Therefore, the resection margins were maintained close to the tumor. However, the margins were positive on histopathological examination, necessitating additional surgery for removal of residual tumor, which was subsequently diagnosed as SC. This discrepancy in clinical and pathological diagnosis might be due to the indolent clinical behavior of SC arising in the minor salivary gland of buccal mucosa. Our case needed to be ruled out for metastases from breast carcinoma since the patient had a history of breast DCIS. The PET-CT did not show any signs of metastases, and the histopathological sections of breast DCIS appeared completely different from the SC in the buccal mucosa. The possibility of metastasis of the breast carcinoma could be completely ruled out.

The differential diagnosis of SC includes AcCC, low-grade cribriform cystadenocarcinoma, low-grade mucoepidermoid carcinoma, and polymorphous low-grade adenocarcinoma [3]. Most of the cases of SC were previously diagnosed as AcCC because of their histopathological similarities. Nevertheless, some histomorphological findings are more common in SC than in AcCC. Few authors reported that the presence of papillary cystic and microcystic patterns with vacuolated cells is characteristic of SC [32, 33]. Hemosiderin deposition was also more commonly observed in SC than in AcCC [34]. In the present case, the absence of zymogen granules and presence of microcystic pattern with eosinophilic cytoplasm and eosinophilic secretory material were suggestive of SC rather than AcCC. Few areas of hemosiderin deposition along with vacuolated cells and the papillary cystic arrangement of cell also favored a diagnosis toward SC rather than AcCC.

The sections stained positive for cytokeratin-19, S100 protein, vimentin, and mammaglobin. S100 and vimentin were strongly expressed as has been reported earlier in SC. Mammaglobin is related to a family of secretory proteins; it is expressed in normal breast cells and overexpressed in carcinomatous breast cells [35]. Strong positive reaction to mammaglobin was noted in the present case, which suggested the presence of a mammary analogue secretory component in the tumor cells. The DOG-1 protein is known to be expressed in normal salivary gland tissues, especially in the apical portions of acinic cells and few areas of the intercalated duct cells [36]. This marker can be utilized to rule out the presence of the acinic component in suspected cases of SC. A negative reaction to DOG-1 was noted in the current case thereby ruling out a diagnosis of AcCC.

The histological, immunohistochemical, and genetic appearance of SC of salivary gland is similar to that of breast secretory carcinoma. A balanced translocation t (12:15) (p13: q25) resulting in ETV6-NTRK3 fusion is seen in SC [2]. Most cases have been confirmed by the demonstration of a break apart or fusion gene by fluorescence in situ hybridization or polymerase chain reaction. However, with increasing numbers of retrospective studies, it was demonstrated that the result of the histomorphologic features and IHC profile was sufficient to diagnose almost all cases of SC, while genetic analysis can be reserved for atypical cases [20, 37, 38].

## 4. Conclusion

This report presents a rare case of SC of buccal mucosa, which was benign in clinical presentation. In addition, a review of the medical literature regarding the clinical behavior of SC of minor salivary gland was performed.

## Figures and Tables

**Table 1:** Reported cases of secretory carcinoma of minor salivary glands and their clinical features.

*Author*	*Site*	*Age/Sex*	*Clinical course (Duration)*	*Size*	*LNI / DM*	*Treatment*	*Follow-up (Duration)*
Abe M, 2015 [5]	Upper lip	61/F	Painless mass (1 year)	0.8	NA	NA	NA

Aizawa T, 2016 [6]	Lower lip	41/M	Slow growth, painless (3 mo)	1.5x1.3	-/-	S	LNM (26 mo)

Bishop JA, 2013 [7]	Buccal mucosa	71/M	NA	1.0	-/-	S	Only 1 case of upper lip locally recurred. Mean follow-up: 42 mo (4-85mo)
	Hard Palate	25/F		NA	-/-	S	
	Soft Palate	20/F		0.9	-/-	S	
	Soft Palate	86/F		1.0	-/-	S	
	Soft Palate	79/F		0.7	-/-	S	
	Upper lip	62/F		0.3	-/-	S	
	Upper lip	71/M		0.6	-/-	S	
	Lower lip	59/M		0.9	-/-	S	
	Lower lip	68/M		0.5	-/-	S	

Bissinger O, 2017 [8]	Floor of mouth	34/M		0.8	-/-	S	No recurrence

Chiosea SI, 2012 [4]	Soft palate-3 case, Buccal mucosa – 2 case, Tongue base- 1 case. Further clinical data not available						

Connor A, 2012 [9]	Oral cavity (NS)	Mean:40 y (14-77y)	NA	1.8	-/-	S	NA
	Roof of mouth			0.5	-/-	S	
	Inner cheek			NA	-/-	S	
	Lip			1.2	-/-	S	

Cooper D, 2013 [10]	Soft palate	43/M	Slow growing, painless	2.5	-/-	S	No recurrence (14 mo)
	Soft palate	26/F	Painless (7 mo)	0.7	-/-	S	No recurrence (24 mo)

Din NU, 2016 [11]	Buccal mucosa	NA					

Fehr A, 2011 [12]	Soft Palate	71/F	NA				
	Oral mucosa	43/M					

Griffith C, 2011 [13]	Upper lip	15/M	NA	NA	-/-	NA	NA

Guilmette J, 2017 [14]	Buccal mucosa	64/F	NA	0.6	-/-	NA	NA

Helkamaa T, 2015 [15]	Hard palate	35/M	Ulcerated, tender (6 mo)	2.0x1.5	-/-	S	No recurrence (18 mo)

Hindocha N, 2017 [16]	Upper lip	27/F	Slow growing (1y)	1.5	-/-	S	No recurrence
	Labial mucosa	51/M	Asymptomatic, firm (3y)		-/-	S	No recurrence

Kai K, 2017 [17]	Buccal mucosa	58/M	NA	3.0	NA	S	No recurrence (1 y)

Keishling M, 2014 [18]	Buccal mucosa	5/F	(4mo)	1.5x1.5x1.3	-/-	S	NA

Khurram SA, 2017 [19]	Buccal mucosa, Lower lip and Soft palate- 1 case each. Further clinical data not available.						

Kratochvil FJ, 2012 [20]	Upper lip	48/M	Slow growth, painless	1.0	-/-	S	No recurrence (8 mo)
	Labial mucosa	52/M	Painless	0.7x0.3	-/-	S	No recurrence (4 mo)

Laco J, 2013 [21]	Upper lip	34/F	Slow growing, painless (2y)	1.5	-/-	S	No recurrence (15 mo)

Luo W, 2014 [22]	Hard palate	41/F	Ulcerated crater	2.0	+/-	S+R	No recurrence (10 mo)

Majewska H, 2015 [23]	Hard palate	54/M	Slow growing, non tender (36 mo), aggressive (2 mo)	2.0x1.0	-/-	S+R	Local recurrence (48 mo), LNM

Mariano FV, 2016 [24] and Projetti F, 2015 [25] each reported 1 case (no site specified). Further clinical data not available.							

Roy S, 2018 [26]	Upper lip	54/M	Slow growing, painless	1.5	-/-	S	NA
	Tongue	22/F	NA	1.5x1.5x1	NA	S	NA

Serrano ML, 2015 [27]	Buccal mucosa	41/F	Slow growing	0.5	NA	NA	NA
	Buccal mucosa	50/M	Slow growing	0.5	NA	NA	NA

Skalova A, 2010 [2]	Buccal mucosa	51/F	Slow growing, painless (1y)	1.0	-/-	S	No recurrence (4 y)
	Soft palate	48/M	Slow growing	1.0x1.5	-/-	S	No recurrence (9y)
	Upper lip	32/M	Painless but grew in size	1.0	-/-	S+R	LNM (86 mo)

Skalova A, 2016 [28]	Buccal mucosa	31/F	NA	1.0	-/-	S	No recurrence (11 mo)
	Buccal mucosa	24/F		1.0	+/-	S	LNM, Local recurrence (2y)
	Upper lip	48/M		1.0	-/-	S	Local recurrence (2 mo)
	Lip	50/M		1.5	NA	NA	NA
	Retromolar gingiva	69/F		0.6	-/-	S	No recurrence (2y)
	Retromolar gingiva	73/M		1.5x2.0x2.5	+/-	NA	NA
	Lip	62/F		1.0	-/-	S	No recurrence (3 y)

Steven TM, 2015 [29]	Upper lip	44/F	NA	NA	NA	S	NA
	Lower lip	66/M	NA	NA	NA	S	NA
	Hard palate	54/M	NA	NA	NA	S+R	No recurrence (2 y)

Urano M, 2015 [30]	Lip	40/M	(3 mo)	1.2x1.1	-/-	NA	No recurrence

Zardawi IM, 2014 [31]	Upper lip	66/M	(6 mo)	1.2x1.0x.08	-/-	S	No recurrence

LNI / DM: Lymph node involvement / Distant metastases before treatment, S: Surgery, R: Adjuvant radiotherapy, LNM: Lymph node metastases on follow-up, mo: Month, y: Year, NA: Data not available, +/-: Present/Absent
